# Using Linkage Analysis to Detect Gene-Gene Interactions. 2. Improved Reliability and Extension to More-Complex Models

**DOI:** 10.1371/journal.pone.0146240

**Published:** 2016-01-11

**Authors:** Susan E. Hodge, Valerie R. Hager, David A. Greenberg

**Affiliations:** 1 Battelle Center for Mathematical Medicine, The Research Institute, Nationwide Children’s Hospital, Columbus, Ohio, 43215, United States of America; 2 Department of Pediatrics, College of Medicine, The Ohio State University, Columbus, Ohio, 43215, United States of America; University of Leicester, UNITED KINGDOM

## Abstract

Detecting gene-gene interaction in complex diseases has become an important priority for common disease genetics, but most current approaches to detecting interaction start with disease-marker associations. These approaches are based on population allele frequency correlations, not genetic inheritance, and therefore cannot exploit the rich information about inheritance contained within families. They are also hampered by issues of rigorous phenotype definition, multiple test correction, and allelic and locus heterogeneity. We recently developed, tested, and published a powerful gene-gene interaction detection strategy based on conditioning *family data* on a known disease-causing allele or a disease-associated marker allele^4^. We successfully applied the method to disease data and used computer simulation to exhaustively test the method for some epistatic models. We knew that the statistic we developed to indicate interaction was less reliable when applied to more-complex interaction models. Here, we improve the statistic and expand the testing procedure. We computer-simulated multipoint linkage data for a disease caused by two interacting loci. We examined epistatic as well as additive models and compared them with heterogeneity models. In all our models, the at-risk genotypes are “major” in the sense that among affected individuals, a substantial proportion has a disease-related genotype. One of the loci (*A*) has a known disease-related allele (as would have been determined from a previous analysis). We removed (pruned) family members who did not carry this allele; the resultant dataset is referred to as “stratified.” This elimination step has the effect of raising the “penetrance” and detectability at the second locus (*B*). We used the lod scores for the stratified and unstratified data sets to calculate a statistic that either indicated the presence of interaction or indicated that no interaction was detectable. We show that the new method is robust and reliable for a wide range of parameters. Our statistic performs well both with the epistatic models (false negative rates, i.e., failing to detect interaction, ranging from 0 to 2.5%) and with the heterogeneity models (false positive rates, i.e., falsely detecting interaction, ≤1%). It works well with the additive model except when allele frequencies at the two loci differ widely. We explore those features of the additive model that make detecting interaction more difficult. All testing of this method suggests that it provides a reliable approach to detecting gene-gene interaction.

## Introduction

### 1.1. The approach

One of the major challenges of human genetics today is reliably determining the existence of gene-gene interactions. Gene-gene interaction has been invoked to explain the so-called “missing heritability,” a phrase coined to help explain the failure of Genomewide Association Studies (GWAS) to account for most of the genetic contribution to clearly genetically-caused diseases. Part of that explanation was that interaction was obscuring GWAS’s ability to find the genetic contribution. The problems encountered in using GWAS data to look for interaction have fostered the development of a number of association-based methods to find interaction. However, GWAS methods face hurdles in detecting gene-gene interactions, including the exponential increase in the number of tests performed.

Accumulated experience (e.g., the Genetic Analysis Workshops) had shown that a linkage analysis can find evidence for a number of loci involved in a disease, but with association analysis it is difficult to determine whether multiple signals represent loci that *interact* to cause disease (epistasis) or loci that contribute *independently* to disease expression (heterogeneity). Furthermore, the nature of gene-gene interaction is such that evidence for interaction, whether association- or linkage-based, can be hidden. In the case of association, interaction can be obscured, by, for example, allelic heterogeneity and locus heterogeneity. In the case of linkage, interaction my be obscured by weak linkage signals at one (or both) of the interacting genes, due perhaps to locus heterogeneity (linkage is robust to allelic heterogeneity). Although careful family-by-family analysis may help clarify such issues [1,2], it is usually difficult to learn whether multiple linkage signals represent interacting loci that cause disease. Hence, the necessity of having a reliable method to determine whether such interaction exists.

In a previous publication [3], we developed a technique for detecting epistatic gene-gene interaction based on linkage analysis, and we applied this technique to data from families with familial primary pulmonary arterial hypertension (FPAH). This led to evidence for a locus, previously undetected, that interacts with the known FPAH causative locus, BMPR2, and explains the greater part of the “reduced penetrance” of the disease for those unaffected family members carrying the BMPR2 mutation. Our approach was based on the principles governing epistatic inheritance, in which disease-related genotypes must be present at both loci in order to manifest the disease.

In later work, we determined the efficiency and effectiveness of the method using computer simulation [4]. We showed that, in the presence of “simple” epistatic interaction, the method had close to 100% efficiency in distinguishing gene-gene interaction from genetic heterogeneity. By “simple” epistasis, we mean traits that were determined by two loci, each of which shows either dominant or recessive inheritance. Moreover, as the FPAH results showed, our approach can also uncover evidence for a previously hidden locus that would be difficult to undetect with a standard approach.

Because the method worked well for simple two-locus epistatic models, we tested it on a more complex model, Additive2 (ADD2). This model requires the existence of two loci, but unlike the EPI models, ADD2 requires that there be a minimum count of 2 disease-related alleles at the two loci in order to cause disease. Thus, the inheritance is neither dominant nor recessive. In the past, we had examined an ADD2 model in the context of detecting linkage [5] and determining gene location [6]. In those works, we saw that, when such underlying inheritance models obtain, one can detect linkage, although the apparent mode of inheritance derived from the linkage data depends on the gene frequencies of the disease alleles at the two loci.

The core of the method we developed requires “stratifying” or “pruning” the data in such a way that only gene carriers of a known causative, or associated, disease-related allele (at the “known” locus) be included in the data. Then the resulting linkage analysis of the pruned dataset is contrasted with the linkage evidence using the unstratified data. In the FPAH case (noted above), the known gene was BMPR2; alleles of that gene have proven to carry disease-causing mutations [7]. In applying our approach, family members not carrying this causative allele were eliminated (or “pruned”) from the data [3], and the analysis of those pruned data showed strong evidence for another locus. Further, the results suggested that presence (or absence) of a disease allele at this second locus was the reason the disease-causing BMPR2 mutations showed only 20% penetrance.

In Corso & Greenberg [4] we developed and tested the *INT* statistic as an indicator of interaction. *INT* is defined as the difference in the maximum multipoint LOD scores between the linkage analysis of the full dataset and the linkage analysis of the stratified, or “pruned”, dataset. We had expected that *INT* would work as well for the ADD2 model. To our surprise, *INT* proved an unreliable indicator of interaction for ADD2. Thus, we wanted to understand why *INT* failed for the case of additive inheritance and to devise a new approach that would retain the reliability of the *INT* statistic but could be successfully applied to a wider range of inheritance models.

### 1.2. Scope of the current project

We have developed a new statistic, designated *INT2*. Our testing indicates that *INT2* yields very few false positives, that is, it rarely indicates interaction when none exists. The false negative rates are low when the data are generated under two-locus epistatic and heterogeneity models, and for the additive2 model when gene frequencies at the two loci are (approximately) equal. Our testing is done under a wide variety of population parameters and different forms of gene-gene interaction, or non-interaction, between two loci. In this report, we describe the development and extensive testing of this interaction indicator. We emphasize that the object of the work is to reliably detect gene-gene interaction. This is not a test of linkage. Our approach assumes there is a known allele at a locus that is a necessary (or at least major), but not sufficient, cause of the disease, and the method tests for interaction between that locus and, for example, another linkage signal. (By “major” genotype we mean that that all affected individuals, or at least the great majority of affected individuals, have the at-risk genotype, or one of the at-risk genotypes, if more than one.)

If the method detects interaction when the true model is heterogeneity, that is a false positive; if it fails to detect interaction when the true model is epistasis, or the additive model, that is a false negative.

## Methods

### 2.1. Generating models

**Fig 1:**
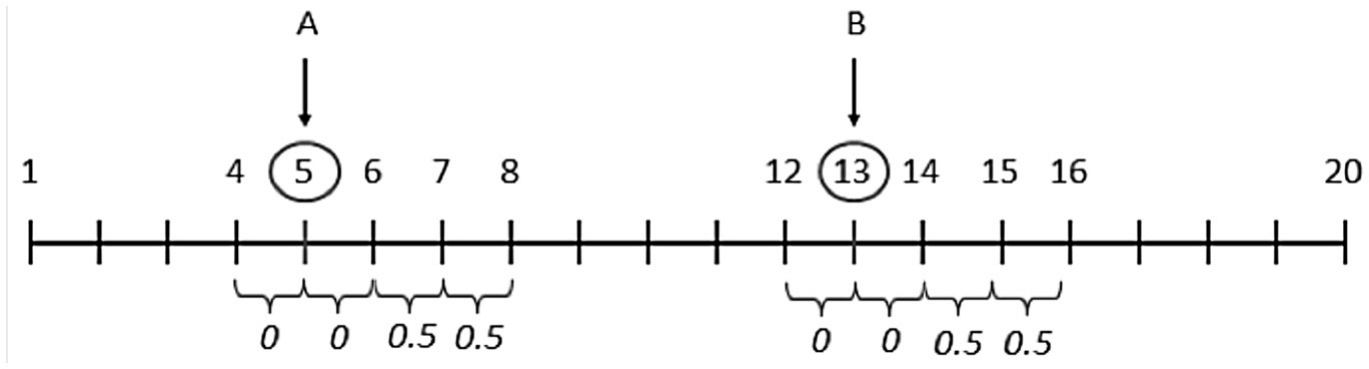
Schematic representation of the “chromosome” used in the simulations. Locus *A* (position 5) is the already known disease locus; locus *B* (position 13) is the test locus. Markers at positions 4 and 6 are linked to *A* with recombination fractions of essentially 0 (*θ* = .001 in the simulations); similarly, markers at 12 and 13 are tightly linked to *B*.

We generated data under several inheritance models (described in Corso & Greenberg [4]). All models were two-locus models, with the loci unlinked to each other. The first disease locus (the “known” locus) is tightly linked to a single marker and the disease allele at that locus is in strong linkage disequilibrium (*r*^2^ = 1) with allele 1 of marker 4 (see Fig 1). Thus, the presence of this marker allele signals the presence of the disease allele at the disease locus, as we explored in Corso & Greenberg [4]. The second locus was the “test” or unknown, locus. The disease allele at the test locus was not in LD with any marker allele. We tested epistatic (EPI), heterogeneity (HET), and additive2 (ADD2) models. We tested different kinds of epistatic interaction between the loci as well as models in which the loci did not interact, i.e., produced disease independently of each other. The values of θ (recombination fraction) are shown in Fig 1.

#### 2.1.1. Genetic structure of the “chromosome”

We generated multipoint linkage data for a disease caused by two unlinked, epistatically-interacting loci (*A* and *B*), or caused by two independent heterogeneous loci, using a modification of the program Caleb [8]. Caleb generates multipoint family data in which two disease loci produce the disease, through interaction or independently (details below). In addition to the two disease loci, the program allows the user to specify up to 18 single nucleotide polymorphisms (SNP) marker loci of arbitrary gene frequency and also allows specification of pairwise linkage disequilibrium (LD) between loci. For all simulations we fixed the first disease locus (locus *A*) at position 5 and the second disease locus (locus *B*) at position 13 on the simulated “chromosome” (see Fig 1). These loci were not linked to each other (recombination fraction (*θ*) = 0.5). We then calculated the LOD score at each position around the test locus *B*.

The genetic distances between loci (i.e., recombination fraction *θ*) used to generate the linkage data are shown in Fig 1. Some of the marker loci between disease locus *A* and disease locus *B* were separated by recombination fractions that ensured *A* and *B* were unlinked. Recombination fractions between pairs of markers directly surrounding the disease loci were fixed at 0.001, approximating *θ = 0* [4].

The LD measure, *D*’, between the disease allele at locus *A* and marker allele 1 at locus 4 was set to 1 and the gene frequency of the disease allele matched that of allele 1; thus, allele 1 at the marker always occurred together with the disease allele (*r*^*2*^ = 1) at locus *A* and never with the normal allele. The recombination fraction between the disease allele at locus *A* and the locus 4 marker was set to 0 (see Fig 1). There was no LD between any other alleles.

The LD block included markers 1 through 6 of the chromosome map. Marker 4, which was used to prune the pedigrees, had the same allele frequency as the disease gene. For all other markers in this section, the allele frequency was fixed at 0.5.

#### 2.1.2. Models

*Epistatic (EPI) models*—We examined four EPI models: dominant-dominant (DD), dominant-recessive (DR), recessive-dominant (RD), and recessive-recessive (RR) (described in detail in Greenberg et al. [9] and Corso & Greenberg [4]). In these models, an individual must have the disease genotype *at both loci* in order to be affected. Moreover, having the disease genotype at both loci is sufficient as well as necessary. Thus, once the genotype is known there is no random component to being affected (although from the viewpoint of either locus alone penetrance appears to be reduced). There were no sporadics. The EPI models are the ones that display interaction.

*Heterogeneity (HET) models*–Heterogeneity exists when the two loci independently cause disease. Inheritance can be either dominant or recessive at either locus. The penetrance at each locus is set to 50%. We refer to the HET models as D+D, D+R, R+D, and R+R, following previously published notation [5]. The HET models do not display interaction.

**Fig 2:**
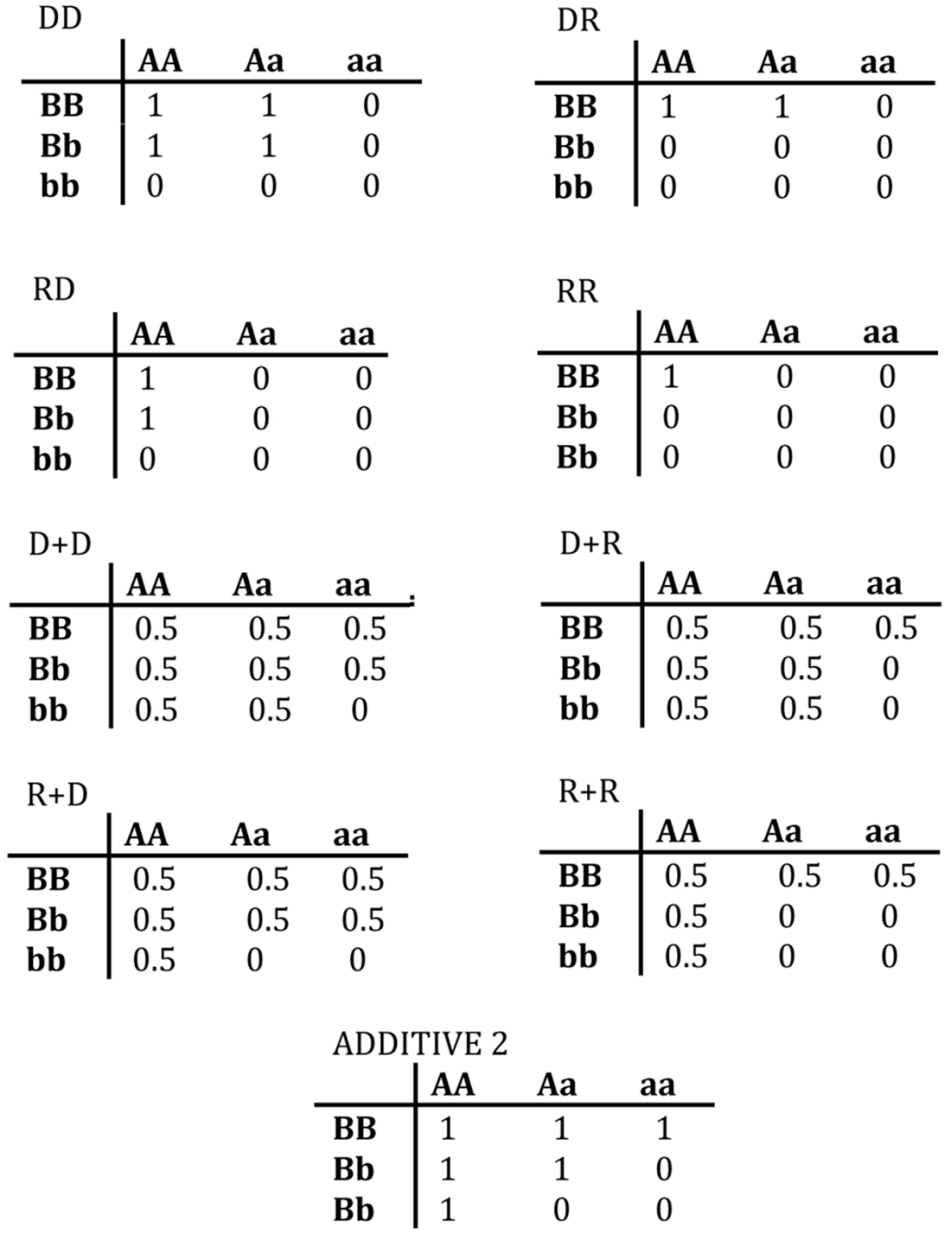
Penetrance structure of the nine two-locus genetic models described in the text. The number in each box gives the penetrance for that genotype, i.e., *P*[affected|genotype].

*Additive Model*–The Additive2 (ADD2) model requires at least two disease alleles, total, at the two loci to cause the trait [5]. Thus this model combines aspects of the DD plus two simple recessive models. Having the disease genotype is sufficient as well as necessary (as with the EPI models). The ADD2 displays interaction, although less so than the EPI models. Fig 2 summarizes the 9 models.

### 2.2. Analyses

#### 2.2.1. Stratification

The core of the method is described in Corso & Greenberg [4]. The method re-structures the family data by “stratifying” or “pruning” the data, eliminating from the analysis those family members who do not carry the disease mutation/associated allele at the known locus. (Connecting individuals, i.e., parents, were always included.) Thus, only gene carriers of the known causative, or associated, disease-related allele (the “known” locus) are included in the data. When interaction exists, the effect of this pruning is to raise the apparent “penetrance” of the disease by eliminating those who cannot be affected because they do not have the disease genotype at one of the necessary/contributing loci.

The datasets were analyzed twice, once before stratification, in which all family members were included, and once after. The linkage results for the two analyses are then used to calculate the statistic *INT*2 (see below), which indicates the presence of interaction.

#### 2.2.2. Simulation parameters

We examined the effects of changing the relative contributions to disease of the *A* and *B* loci. For the HET models, we set penetrance to 0.5 and disease prevalence to .05. We defined *r* as the ratio of *A*’s prevalence contribution to *B*’s prevalence contribution. For the EPI models, prevalence was .01 and penetrance was full. Here *r* was defined analogously to the definition for the het models. For the ADD models, prevalence was set at an intermediate value of .03, penetrance was full, and we defined *r* as the ratio of *A*’s allele frequency to B’s allele frequency. See S1 Appendix for the formulas. We compared results for *r* = .5, 1, 2 in all cases.

We also experimented with dataset sizes of 20, 50, 100, and 200 nuclear families. We simulated two-generation families with a minimum of 2 offspring, and at least one affected offspring. The family size distribution followed that of Cavalli & Bodmer [10] Each simulation consisted of 1,000 datasets.

#### 2.2.3. Analysis parameters

The same genetic map was used for the simulation and the analysis. The analysis gene frequency was assumed to be that of the second locus, locus *B* (the “test” locus). GENEHUNTER was used to compute LOD and HLOD (heterogeneity lod) scores [11] Each dataset was analyzed assuming both dominant and recessive modes of inheritance, at assumed penetrances of 0.5 [12] [13], for both HLOD and LOD scores.

### 2.3. Test statistic *INT2*

The previously described test statistic *INT* [4] had been calculated for each dataset as

INT=(max stratified multipoint LOD)−(max unstratified multipoint LOD)  (1)

When we found that *INT* produced too many false negatives for the ADD models and too many false positives for some of the het models, we devised a new statistic, *INT2*. To begin, we maximized the LODs over the two analysis models, dominant with 50% penetrance and recessive with 50% penetrance (MMLS = maximized max lod score). Define

$$\begin{array}{l} U=\text{max unstratified MMLS multipoint LOD}\\ S=\text{max stratified MMLS multipoint LOD}\\ INT^{\prime }=S-U \end{array}$$ (2)

This *INT*′ (“*INT* prime”) is analogous to our original *INT* statistic as in Eq (1), but uses MMLS. However, experience shows that the statistic still needs “scaling.” We determine whether *S* has the same sign as the just-defined *INT’* or not. When yes, *INT’* is multiplied by (2 + *S*)/U; otherwise, *INT’* is multiplied by (2 –*S*)/U. Thus:

$$INT2=\left\{ \begin{array}{l} \frac{INT^{\prime }\cdot (2+S)}{U}\;(INT^{\prime }\;and\;S\;have\;same\;sign)\\ \frac{INT^{\prime }\cdot (2-S)}{U}\;(INT^{\prime }\;and\;S\;have\;opp.\;signs) \end{array}\right.$$ (3)

We also calculated the stratified max MMLS *HLOD* (called *S*_*het*_). We used this score for initial screening: We eliminated datasets for which *S*_*het*_ was less than 1 and did not include them in further analysis (see Sec. 3.1). Note that for eliminating datasets we used the HLOD, but for the test statistic *INT2* we used the LOD score without heterogeneity, as in Eq (2).

By definition, datasets that survived this initial screen (*S*_*het*_ > 1) and whose subsequent *INT2* score was greater than or equal to zero are interpreted as giving evidence of interaction.

### 2.4. Summary of test procedure

To calculate *INT2*:

Step 1. Eliminate datasets with insufficient information: Determine MMLS HLOD scores [6] for the stratified version of each dataset, and discard/eliminate those datasets with (*S*_*het*_ < 1). Experience showed that applying this cutoff reduced the number of false positives. (In Corso & Greenberg [4], we had used a cut-off of stratified LOD < 1.5.)
Step 2. Estimate mode of inheritance: Calculate the MMLS scores (using the LOD, not HLOD) for the unstratified datasets that survived Step 1. The LOD score at the test locus for each unstratified version was maximized with respect to mode of inheritance. That mode of inheritance that led to the higher LOD score was then used to (re-)calculate the LOD score for the stratified version of each dataset.
Step 3. Compute stratified (*S*) and unstratified (*U*) max MMLS LOD scores, and use them to calculate *INT*′ and *INT2*, as in Eqs (2) and (3).

For each simulation, the distribution of the statistic *INT2* was then plotted as a histogram, in increments of 0.2 on the *INT2* axis, in the range [–10, 15]. Occasionally some values fell outside that range, which was noted and recorded.

## Results

### 3.1. The *S*_*het*_ > 1 cutoff criterion

In extensive simulations, many datasets generated under the HET and ADD models yielded unacceptably high error rates. Most frequently, the datasets that led to false positives or false negatives were those in which the stratified MMLS HLOD score (*S*_*het*_) was less than 1 (Sec. 2.3). Thus, as described in Methods, we first determined whether a dataset’s *S*_*het*_ was greater than 1. Table 1 shows that no EPI datasets, and only few ADD2 datasets, failed to met this *S*_*het*_ criterion, but that failure was very frequent for the HET datasets (between 95.5% and 97% of datasets for the 4 het models we examined). Thus it seems reasonable to interpret *S*_*het*_ < 1 as representing *prima facie* evidence against interaction. We return to this point in the Discussion.

**Table 1:** Distribution of *INT2* values, for all 9 models. The table shows the number of datasets, out of 1,000, in each category.

**Epistatic models**			
**Model**	***S***_***het***_ **< 1**	***INT2* < 0 (error rate)**	***INT2* > 0**
**DD**	**0**	**0 (0%)**	**1,000**
**DR**	**0**	**2 (0.2%)**	**998**
**RD**	**0**	**4 (0.4%)**	**996**
**RR**	**0**	**25 (2.5%)**	**975**
**Heterogeneity models**			
**Model**	***S***_***het***_ **< 1**	***INT2* < 0**	***INT2* > 0 (error rate)**
**D+D**	**959**	**41**	**0 (0%)**
**D+R**	**955**	**39**	**6 (0.6%)**
**R+D**	**971**	**25**	**4 (0.4%)**
**R+R**	**970**	**20**	**10 (1.0%)**
**Additive model**			
**Model**	***S***_***het***_ **< 1**	***INT2* < 0 (error rate)**	***INT2* > 0**
**Add2**	**1**	**46 (4.6%)**	**953**

*r* = 1 for all entries in this table.

Dataset size = 100 families/dataset

Number in each cell gives the number of datasets out of 1,000 datasets (% in parentheses).

***S***_***het***_ = max stratified MMLS HETLOD (Sec. 3.1)

### 3.2. Comparing *INT2* to *INT*

**Fig 3:**
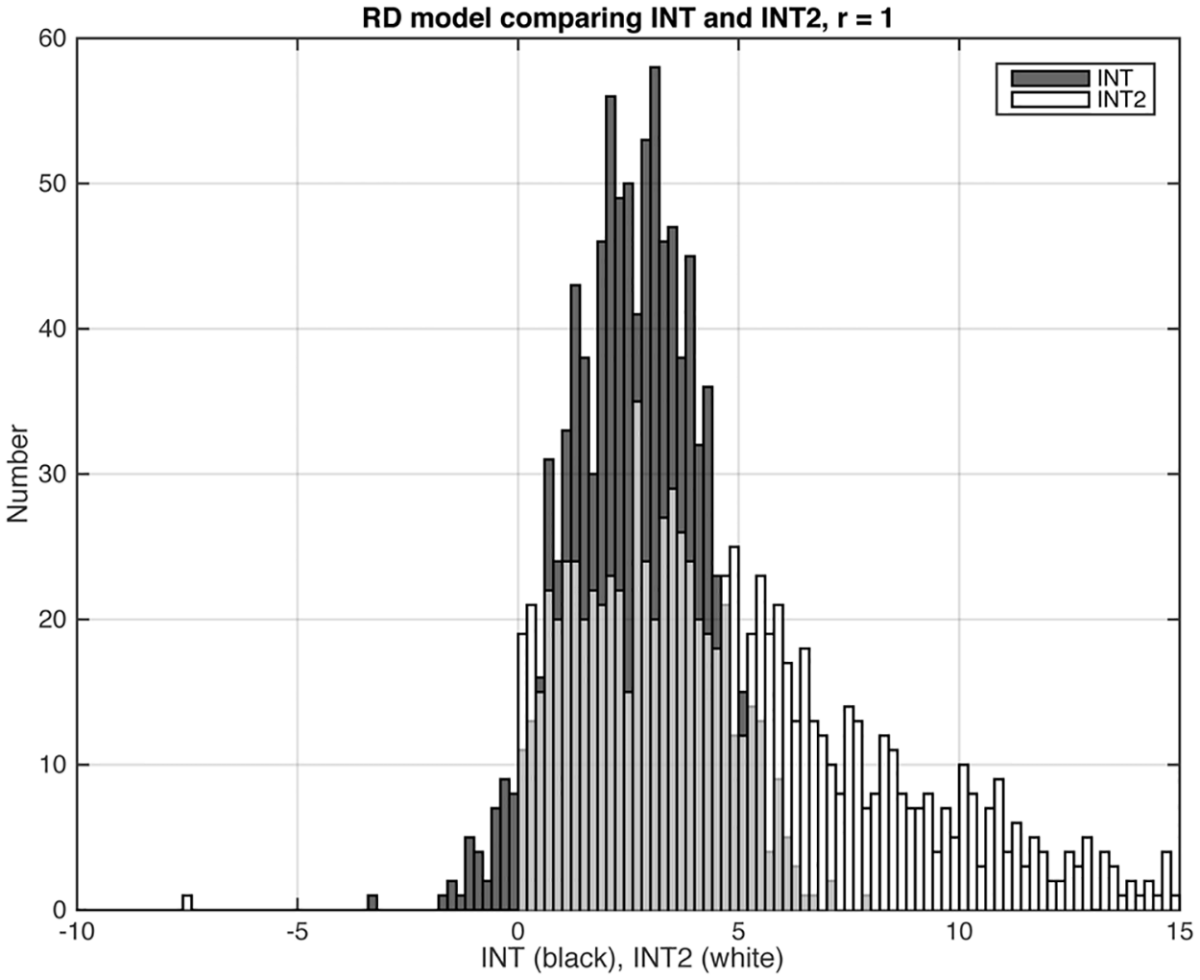
Distributions of *INT* and *INT2* for the RD model, among the datasets that met the *S*_*het*_ > 1 cutoff criterion (Sec. 3.1). For the EPI models, the statistic should have positive values. The ratio *r* equals 1, and the graph shows results from 1,000 datasets. *INT* and *INT2* values that were < –10 or > 15 do not show in the graphs but are noted here: For *INT*, all datasets are included in the graph; for *INT2*, 3 datasets had values < –10; 48 had values > 15.

**Fig 4:**
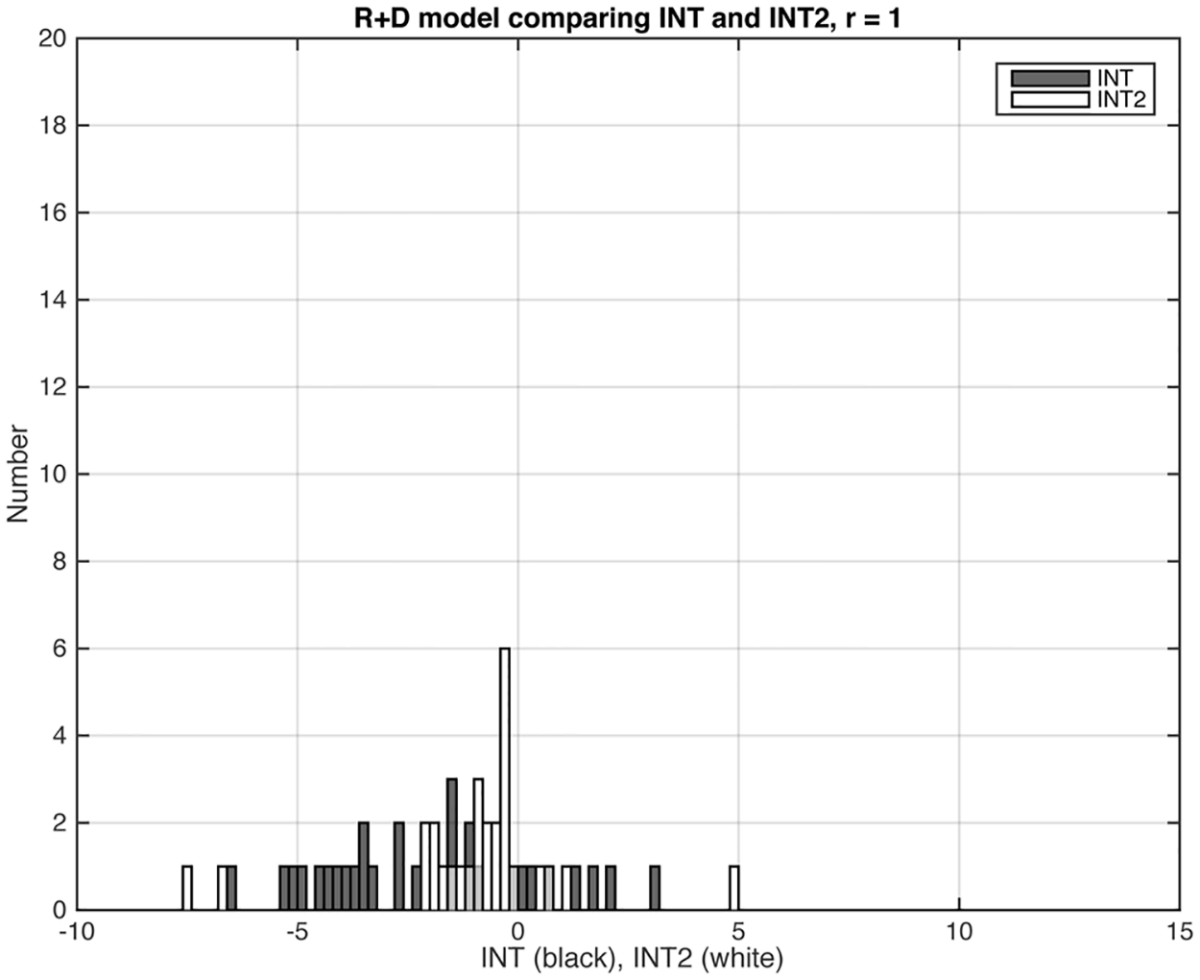
Distributions of *INT* and *INT2* for the R+D model, among the datasets that met the *S*_*het*_ > 1 cutoff criterion (Sec. 3.1). For the HET models, the statistic should have negative values. The ratio *r* equals 1, and the graph shows results for 29 datasets. *INT* and *INT2* values that were < –10 or > 15 do not show in the graphs but are noted here: For *INT*, all datasets are included; for *INT2*, 1 dataset had a value < –10.

**Fig 5:**
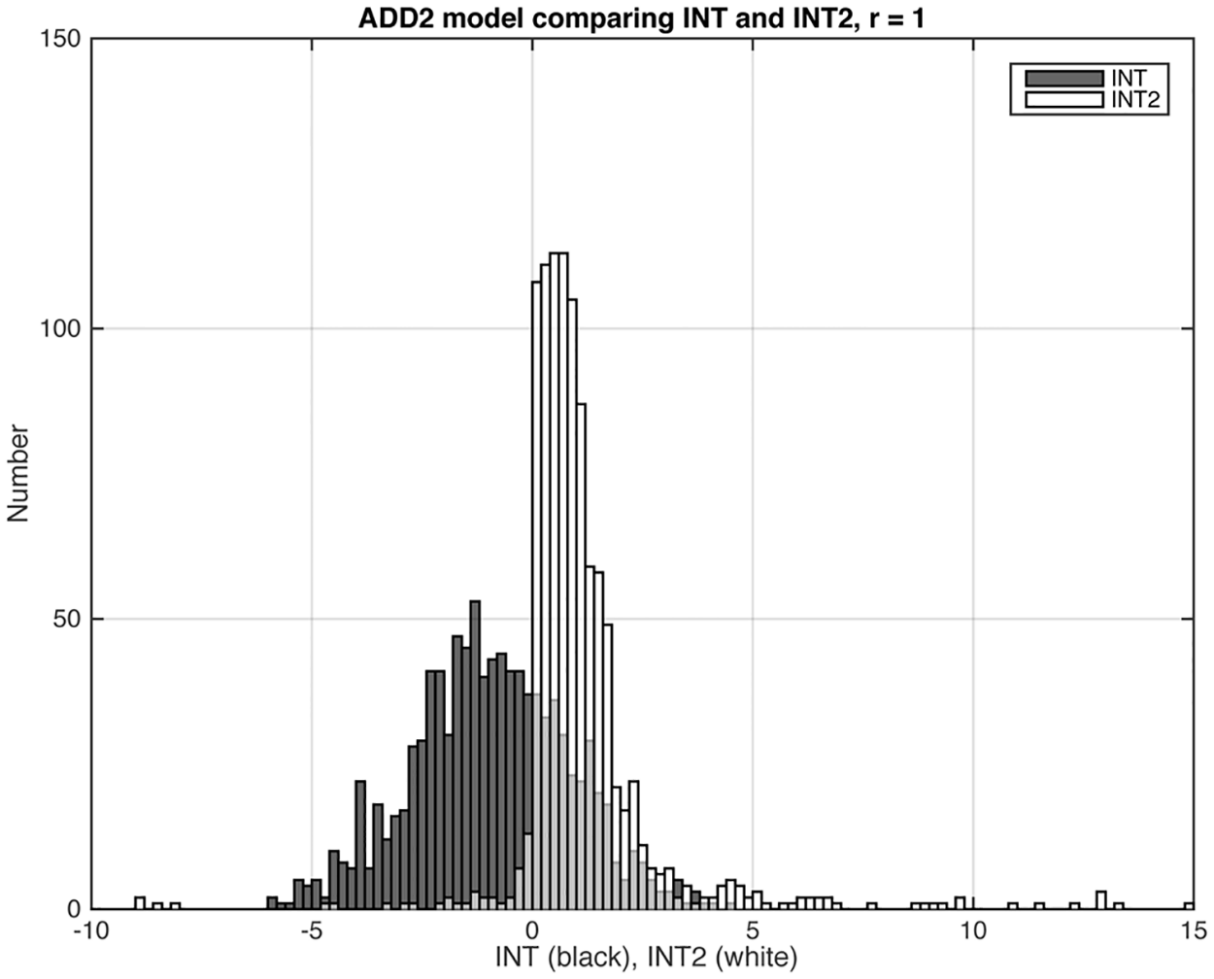
Distributions of *INT* and *INT2* for the ADD2 model, among the datasets that met the *S*_*het*_ > 1 cutoff criterion (Sec. 3.1). For the ADD2 model, the statistic should have positive values. The ratio *r* equals 1, and the graph shows results for 999 datasets. *INT* and *INT2* values that were < –10 or > 15 do not show in the graphs but are noted here: For *INT*, all datasets are included; for *INT2*, 2 datasets had values < –10, and 3 had values > 15.

In numerous simulations, we determined that *INT2* consistently yielded error rates that were at least as low as those from *INT* for all models, and were much superior to those from *INT* for the ADD models and for D+R. We illustrate with the distributions of *INT* and *INT2* for three representative models (Figs 3, 4 and 5). Figs 3 and 5 show results for, respectively, one of the EPI models (RD) and one of the ADD models (ADD2). These are models where the statistic should be positive because the models have gene-gene interaction. (In the case of the ADD2 model, the extent of positivity depends on the gene frequency of the disease alleles at the two loci; see below.) In both cases, *INT2* has a more positive distribution than *INT*. This difference is most striking for the ADD2 model, where *INT* was overwhelmingly negative and *INT2* is primarily positive. Fig 4 shows results for one of the Het models (R+D), where we want the statistic to be negative. Only 29 out of 100 datasets satisfied the *S*_*het*_ cutoff criterion. Of these, *INT* yielded 8 false positives and *INT2* only 5. Since *INT2* was consistently as least as reliable as *INT* overall, we report only *INT2* for the remainder of this study.

### 3.3. Effects of relative allele frequencies

The gene frequency effects for the EPI and HET models were relatively minor and did not qualitatively affect the ability of INT2 to detect interaction. The ADD models, in contrast, were sensitive to the ratio *r* described above (Sec. 2.2.2). We address this observation in the Discussion.

**Fig 6:**
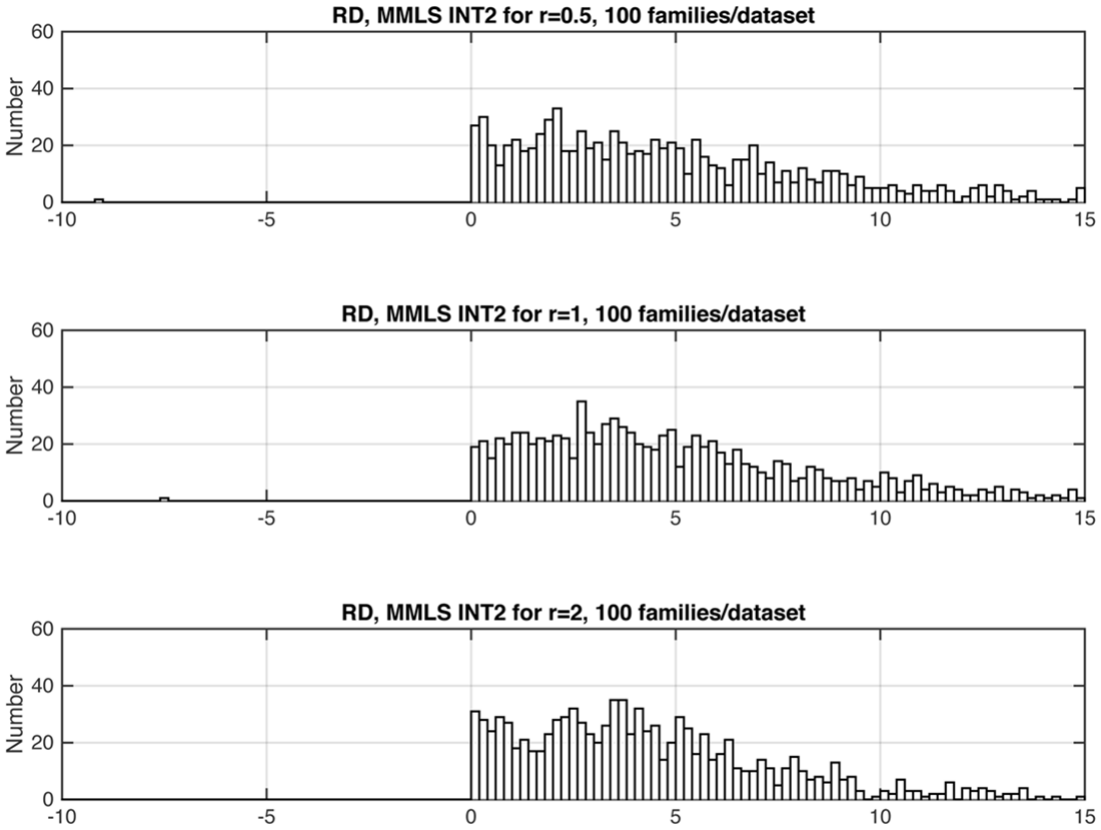
For the RD model, distribution of *INT2*, as a function of *r* (see Sec. 2.2.2 and Tables B and C in S1 Appendix). For the EPI models, the statistic should have positive values. There were 1,000 datasets for all values of *r*. *INT2* values that were < –10 or > 15 do not show in the graphs but are noted here: For *r* = .5, 90 datasets had values > 15; for *r* = 1, 3 had values < –10, and 48 had values > 15; for *r* = 2, 22 datasets had values > 15.

**Fig 7:**
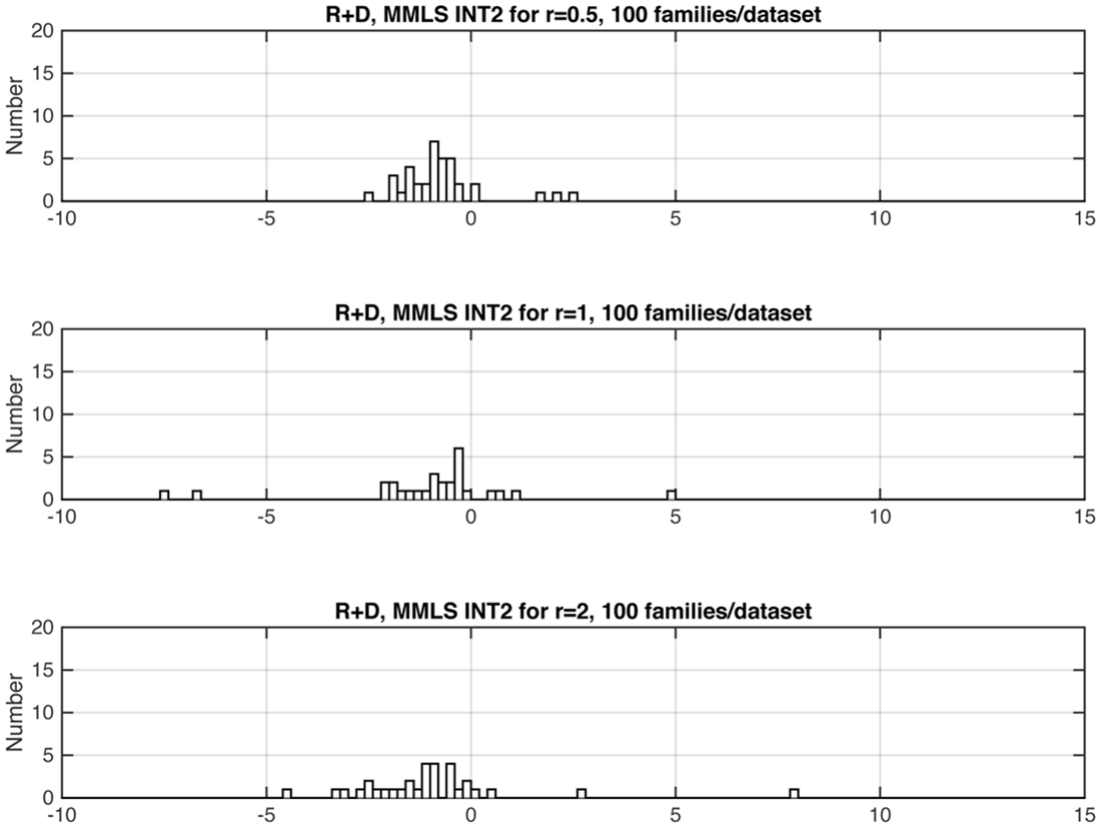
For the R+D model, distribution of *INT2*, as a function of *r* (see Sec. 2.2.2 and Tables A and C in S1 Appendix). For the HET models, the statistic should have negative values. *INT2* values that were < –10 or > 15 do not show in the graphs but are noted here: For *r* = .5, there were 37 datasets; for *r* = 1, there were 29 datasets, of which one had a value > 15; for *r* = 2, there were 33 datasets.

**Fig 8:**
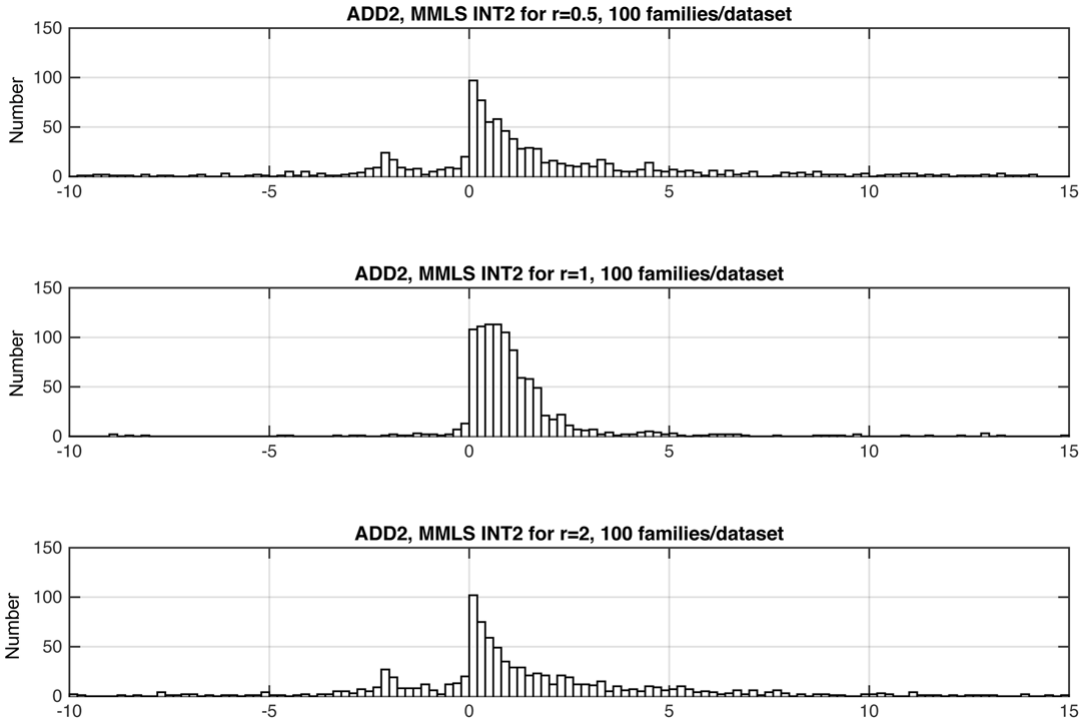
For the ADD2 model, distribution of *INT2*, as a function of *r* (see Sec. 2.2.2 and Table C in S1 Appendix). For the ADD2 model, the statistic should have positive values. *INT2* values that were < –10 or > 15 do not show in the graphs but are noted here: For *r* = .5, there were 991 datasets, of which 40 had values < –10 and 39 had values > 15; for *r* = 1, there were 999 datasets, of which 2 had values < –10, and 3 had values > 15; for *r* = 2, there were 989 datasets, of which 42 had values < –10 and 39 datasets had values > 15.

Figs 6, 7 and 8 shows three representative examples of the effects of changing relative gene frequencies on the distribution of INT2. For the rest of the study we report results with *r* = 1. S1 Appendix shows the allele frequencies corresponding to *r* = 1, for all models examined.

### 3.4. *INT2* results

Table 1 gives the error rates for *INT2*, for all the models we examined. We define error rate as the proportion of datasets, out of 1,000, that supported interaction when there was none (false positives), or that failed to support interaction when interaction was present (false negatives). Thus, for datasets generated under HET models, positive *INT2* scores count as false positives, whereas for the EPI and ADD models, negative *INT2* scores are false negatives. The table records first how many datasets had *S*_*het*_ < 1 and were therefore eliminated from further analysis (see Sec. 3.1). Among the remaining datasets, we tabulated whether *INT2* was positive (supporting interaction) or negative (not supporting interaction).

For the EPI and ADD models, essentially no datasets were eliminated in the initial screen (i.e., there were no datasets with *S*_*het*_ < 1), and for the EPI models, error rates (*INT2* < 0, false negatives) were low, ranging from 0 to 2.5%. For the ADD model, the situation is more nuanced. Error rates were good (0.5% to 4.6%) when the allele frequencies at the two loci were equal. But these errors rose sharply as the two allele frequencies diverged. See Discussion (Sec. 4.3).

For the het models, over 95% of the datasets had *S*_*het*_ < 1, and most of the remaining datasets had *INT2* < 0. Both of these outcomes support lack of interaction; only *INT2* > 0 supported interaction, leading to error rates of less than 1% in our simulations.

## Discussion

### 4.1. About *INT2*

An ideal interaction test statistic should yield positive values for the EPI models and negative values for the HET models. For the ADD models, it should also yield positive values, although not as robustly as with the EPI models; we base this criterion/requirement on the fact that the ADD models exhibit interaction, but less straightforwardly than the EPI models do. The original statistic, *INT* in Corso & Greenberg [4], was straightforward and intuitively appealing, and it met these criteria for most of the EPI and HET models. However, it performed poorly for ADD2. We wanted a statistic that could detect interaction *even in the presence of heterogeneity*, since the presence of both interaction and heterogeneity is a distinguishing feature of ADD2 (see Sec. 4.3). With some fine-tuning, we developed *INT2* in Eq (3).

### 4.2. Simulation conditions (informativeness, prevalence, gene frequency, dataset size)

Concerning changing the gene frequencies (Sec. 3.3): We fixed the prevalences (0.01 for the EPI models, 0.03 for the ADD models, and 0.05 for the HET models) and varied only the relative frequencies at the two loci. We had performed extensive simulation studies exploring gene frequency, variations of inheritance models, cut-off values, using LOD vs. HLOD, etc. (details not shown). We found that changing the disease prevalence within a reasonable range (prevalence less than 10%) had little effect on the results, except that simulations take longer when low prevalences are simulated (because the simulator creates entire populations that are then selected according to ascertainment criteria set by the user).

Setting a minimum HLOD value (*S*_*het*_ > 1) before calculating *INT2* eliminated most false positive and many false negative results.

Table 2 shows the effect of dataset size on identifying the existence of interaction for several representative models. (Models not shown gave similar results.) It is striking that for the DD model, even a dataset of only 20 families has a high probability (>98%) of showing evidence for interaction. The RD model has a relatively high rate of false negatives (*INT2* < 0) for a dataset size of 20 families (14%), but that drops to <3% with 50-family datasets, and essentially to zero for 100-family datasets. The ADD2 model is more dependent on large samples to be reasonably sure of detecting interaction. For the ADD2, only with 100-family datasets does the false negative rate drop below 10%; by 200 families/dataset, the false negative rate essentially goes to zero. Interestingly, the heterogeneity false positive rates appear not to depend on sample size; all hover at around 1–2%.

**Table 2:** Number of datasets with and number of datasets yielding false positives (for 2 representative HET models) or false negatives (for 2 representative EPI models and the ADD2 model), as a function of dataset size.

		No. of families/dataset			
Model		20	50	100	200
**D+D**	*S*_*het*_ > 1	37 (3.7%)	36 (3.6%)	41 (4.1%)	37 (3.7%)
	False positives (INT2>0)	8 (0.8)	9 (0.9)	0 (0)	0 (0)
**DD**	*S*_*het*_ > 1	992 (99.2)	1,000 (100)	1,000 (100)	1,000 (100)
	False negatives (INT2<0)	17 (1.7)	0 (0)	0 (0)	0 (0)
**R+D**	*S*_*het*_ > 1	36 (3.6)	34 (3.4)	29 (2.9)	27 (2.7)
	False positives (INT2>0)	7 (0.7)	13 (1.3)	4 (0.4)	10 (0.1)
**RD**	*S*_*het*_ > 1	890 (89.0)	998 (99.8)	1,000 (100)	1,000 (100)
	False negatives (INT2<0)	137 (13.7)	27 (2.7)	4 (0.4)	0 (0)
**ADD2**	*S*_*het*_ > 1	563 (56.3)	920 (92.0)	999 (99.0)	1,000 (100)
	False negatives (INT2<0)	260 (26.0)	223 (22.3)	46 (4.6)	1 (0.1)

Number in each cell gives the number of datasets out of 1,000 datasets (% in parentheses).

*r* = 1 for all entries in this table.

***S***_***het***_ = max stratified MMLS HETLOD (Sec. 3.1)

### 4.3. The ADD2 model

**Fig 9:**
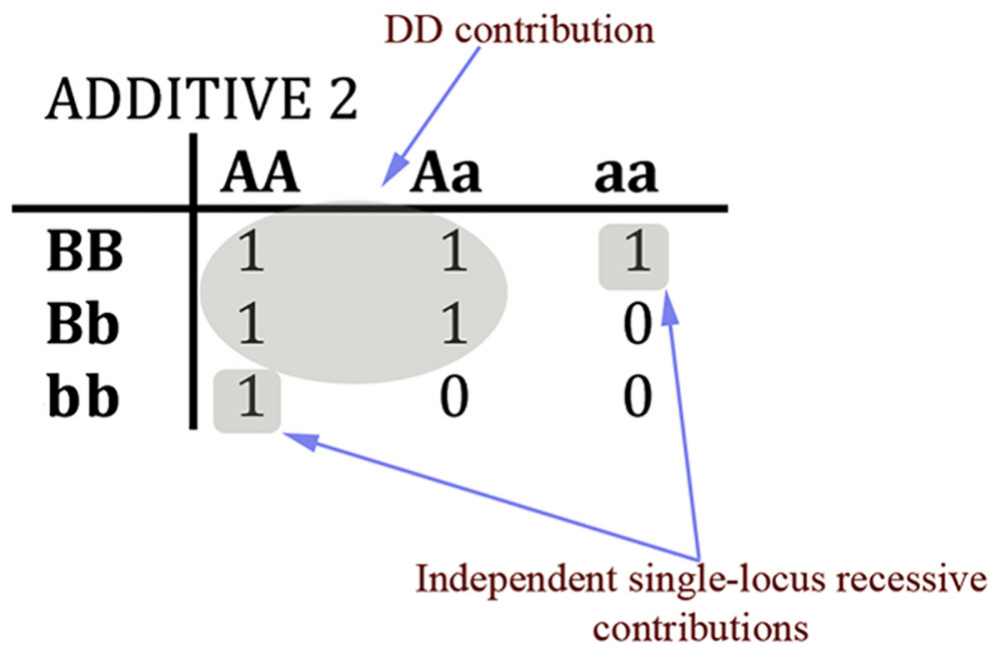
Diagrammatic representation of the ADD2 model, highlighting the DD contribution and the two single-locus recessive contributions.

Why does the ADD models represent interaction but less strongly than the EPI models? One way to understand this is to examine the structures of the three types of models, as in Fig 2. The EPI models have a strictly rectangular (multiplicative) penetrance structure, and the HET models represent the superposition of two separate rectangles, whereas the ADD2 model has a “diagonal” structure that falls between those of the EPI and HET models. The ADD2 model can also be viewed as a composite with both epistatic and heterogeneous components. That is, ADD2 can itself be considered a kind of heterogeneity model, in that it is a mixture of DD and two *single-locus* R models (Fig 9). As a result, as the disease allele frequency at either locus increases, the data become more “single-locus”-like, and more and more of the datasets show negative *INT2*. Also see the next section (4.4).

### 4.4. *INT2*’s effectiveness in detecting interaction

The procedure we developed involves rejecting those *stratified* datasets with *S*_*het*_ < 1 (Sec. 3.1) and then determining whether *INT2* > 0. Removing the *S*_*het*_ < 1 datasets from consideration eliminates many false positive and false negative *INT2*s; specifically, for the HET models, the *S*_*het*_ > 1 criterion appears, by itself, to also be useful as an indicator of interaction. The ADD2 model is a complex model, with both a DD form and two *single-locus* recessive forms of disease contributing to the patient population (see Sec. 4.3), and the DD form is a gene-gene interactive model. We have seen (Table 1) that in the presence of clear epistasis or clear heterogeneity, *INT2* almost always yields a correct result, i.e., detects interaction for EPI models and fails to detect interaction for HET models.

However, the results with ADD2 were more ambiguous. We asked, How reliably can *INT2* detect interaction, in the presence of both gene-gene interaction and heterogeneity, as represented by the ADD2 model? To answer this question, we broke down ADD2 datasets by the number of families that had affected members exclusively caused by the first recessive form, i.e., where the *A* locus is acting recessively; exclusively the second recessive form, i.e., where the *B* locus acts recessively; exclusively the DD form; or mixed, containing (at least) two forms. We also varied the disease allele frequencies to get a range of interaction detection.

Out of 100,000 ADD2 families with *r* = 1, 66% had the DD form and 30% had one of the R forms (only 4% of the families were mixed). Among the 1,000 datasets containing these families, the DD interaction was detected (*INT2* > 0) in 96% of them. When *r* = 5, only 41% of the families had members with the DD form, whereas 56% had an R form (3% were mixed). Even when only 41% of families displayed the DD form, *INT2* was able to detect interaction in 15% of the datasets, suggesting some power to detect interaction even in the presence of substantial heterogeneity. Thus, even though the contribution of the families exhibiting interaction dropped from 66% to 41%, *INT2* could detect interaction in a notable proportion of the datasets, even in a heterogeneous background of single-locus recessive models.

### 4.5. Why focus on linkage-based methods?

In the post-GWAS age of exome and whole genome sequencing, the obvious question arises: Why are linkage-based methods important? We compare aspects of association analysis and linkage analysis (Sec. 4.5.1), and then apply them specifically to this study (Sec. 4.5.2).

#### 4.5.1. Differences between linkage methods and association analysis

These two types of analysis have different designs, use different types of data, and answer different questions. Linkage analysis uses family data, thus taking advantage of the rich genetic information contained in the inheritance patterns within families. It determines whether alleles at a locus *cosegregate* with disease, meaning that some allele at that locus consistently gets inherited along with the disease. (Which allele it is does not matter and may not be the same in different families; what matters is that the cosegregation is consistent within a family.) Association analysis, in contrast, uses data from unrelated individuals in the population. It can determine whether a given allele or SNP is associated with the disease in the population. For association analysis, the allele is the focus of interest, and thus it does matter which allele is which. Because of their design, association methods have high sensitivity for detecting alleles that may have only a minor effect on disease expression (i.e., that increase disease susceptibility only slightly), but low specificity for determining whether a detected allele has a major effect and is worth pursuing. See examples in the next section (4.5.2).

Linkage analysis, on the other hand, has high specificity but relatively low sensitivity [14]. Linkage analysis does not detect susceptibility loci that increase disease risk only slightly. Any gene detected using linkage is virtually certain to have a major influence on disease expression and is, therefore, worth the effort of identifying and pursuing in order to understand the origins of the disease.

Association analysis is vulnerable to both allelic and locus heterogeneity. Allelic heterogeneity refers to a situation in which different alleles at the same locus can produce the same trait or disease, including both classical examples, such as the β-thalassemias and phenylketonuria, and more recent ones, such as our work with FPAH [3]. Since association analysis compares population frequencies between cases and controls, the existence of multiple alleles weakens evidence for an effect.

In contrast, linkage analysis, by definition, pays no attention to the *identity* of marker alleles; it looks only at whether the marker alleles within each family cosegregate consistently with the disease. Consider a disease (let it be single-locus for ease of illustration) where alleles “1” and “2” can each, independently, cause the disease. Some families may segregate the “1” allele and others, the “2” allele. In each case, linkage looks only at the cosegregation of that allele with the disease in that family; whether that allele is the “1” or the “2” is irrelevant to a linkage analysis.

Association analysis faces some additional hurdles. (1) It is also sensitive to locus heterogeneity, whereas linkage analysis has methods to compensate for and even detect locus heterogeneity [6] [13] [15]. (2) When it comes to studying interaction, association methods can detect only joint allele frequency differences between collections of unrelated individuals. Such differences may be only loosely related to inheritance and therefore only loosely correlated with gene-gene interaction. They can demonstrate correlation but not causation. Also see examples in the next subsection. (3) Phenotypes: Because association studies need very large samples in order to detect minor allelic effects on disease expression, they necessarily cannot be as rigorous about phenotype definition as linkage studies are.

#### 4.5.2. Issues specific to our study

In this project we have worked solely with models in which the at-risk genotypes play a major role in disease causation. By “major role” we mean that a substantial proportion of affected individuals have (one of) the at-risk genotype(s). For example, define P as the proportion of affected individuals with the at-risk genotype. For the models shown in Fig 2, this proportion P is 100%, since the penetrances of all the not-at-risk genotypes are zero. Increasing those latter penetrances only slightly may have a large effect on P, since the not-at-risk genotypes are much more frequent in the population than the at-risk genotype(s). For example, with our dominant-dominant (DD) model, raising all the not-at-risk genotype penetrances from 0 to 0.01 reduces P to 50%. In essence, such a change reduces the *genetic* contribution to the disease. P may serve as a rough tool to characterize models for which the at-risk genotype(s) play a major role.

These considerations lead to an interesting question: For the purpose of detecting gene-gene interaction, what is the relationship between genetic models that lend themselves to linkage analysis and genetic models detectable by association analysis? Or, in operational terms, what is the relationship between genotype penetrances, on the one hand, and odds ratios (ORs) from case-control studies on the other? Consider this idealized case-control table, applied to our DD model. (*A* and *B* are the disease alleles at the first and second locus, respectively; *a* and *b* are the normal alleles; *AX = AA* or *Aa*; *BX = BB* or *BB*; *aa–* = homozygotes for *a* without regard to genotype at the *B* locus; the *p*_*i*_ are the genotype frequencies among cases; the *q*_*i*_ are the genotype frequencies among controls.) Table 3 shows the setup.

**Table 3:** Setup of case-control study.

	*AXBX*	*AXbb*	*aa–*	Total
Cases	p_1_	p_2_	p_3_	1.000
Controls	q_1_	q_2_	q_3_	1.000

Define the marginal OR for *A* as $\frac{(p_{1}+p_{2})q_{3}}{(q_{1}+q_{2})p_{3}}$ and the interaction OR as $\frac{p_{1}(q_{2}+q_{3})}{q_{1}(p_{2}+p_{3})}$. Our epistatic models in Fig 2, with all penetrances equaling 1 or 0, lead to ORs of infinity. More interesting is to see what happens when we lower the 1s and raise the 0s. For example, consider a DD model (allele frequencies equal 0.1 for both the *A* and *B* alleles), where we reduce the 1s in Fig 2 to 0.9 and increase the 0s to 0.01. This results in a disease population prevalence of ~2%, and of those affected, ~48% have one of the *AXBX* genotypes (i.e., P = 0.476). This model is easily handled by our linkage-based methods. It would also be readily detectable by comparing ORs, since the marginal ORs for both the *A* and *B* loci would equal 10.9, and the interaction OR would be 891.

In contrast, consider a model where we reduce the 1s in Fig 2 to 0.4 and raise the 0s to 0.1. The ORs still support interaction: marginal OR = 1.3, interaction OR = 6.0. But now not only is the disease more common (population prevalence ~10%), but of those affected, only 3.9% have one of the *AXBX* genotypes; 8.7% have *AX* but not *BX*; another 8.7% have *BX* but not *AX*; and 78.7% do not carry either disease allele *A* or *B* at all. Thus, this is more of a heterogeneity, or even nongenetic, model than an interaction model: Many more affected individuals carry either *A* or *B* alone than carry both *A* and *B*, and, further, the overwhelming majority of affected individuals do not carry either disease allele at all. This example illustrates what we said above, that association analysis is more sensitive, yet what it detects may not be particularly helpful for understanding disease causation.

Holzinger et al. [16] provide a more extreme example, similar to our RR model, with the 1 in Fig 2 lowered to .959, and the zero penetrances raised to values ranging from a minimum of 0.01 to a maximum of .382, for the different genotypes. Both disease allele frequencies equal 0.4. The marginal ORs for A and B both equal ~1, but the interaction OR is 60.5; i.e., the ORs for each of the individual disease alleles show *no* marginal effect, yet there is a strong interaction effect. This hypothetical disease has a population prevalence of 29.5%, that is, almost one-third of the population has the disease. Of these affected individuals, only 8.3% have the interaction genotype (*AABB*); 7.6% are homozygous for *A*; 7.7% are homozygous for *B*; and the majority of affected individuals, 76.3% are not even homozygous for either. Again, this is no longer really an interaction model, but closer to a heterogeneity model, with the majority of cases being nongenetic.

In summary, when the genes are of major effect, linkage methods are powerful for detecting the those genes and, as we have shown, for detecting interaction, but linkage analysis is not suited for models with very low P. Association analysis, in contrast, is able to detect diseases in which the interaction OR is greater than the marginal ORs, whether P is high or very low. However, even though interaction is detectable by association analysis, for diseases with very low P, interaction may not play much of a role in disease etiology. Additionally, association analysis presents other problems. To begin, the investigator would likely test the one million or so available SNPs, correcting for the multiple tests, in order to find those SNPs with significant marginal ORs. Then, assuming the protocol required testing for interaction only among the number *N* that were significant, test the *N*(*N*+1)/2 possible pairwise combinations for interaction ORs. (For example, if only a tiny fraction of the first tests were significant, say *N* = 1,000, one would then need to test ~500K pairs, again dealing with the multiple test problem.) This hypothetical association analysis would also be hampered by the issues mentioned above, of phenotype definition, allelic heterogeneity, and locus heterogeneity. Moreover, those diseases detectable by association analysis but not linkage analysis tend to be precisely those for which the disease alleles are just susceptibility alleles, so that even once they are detected, we may be no closer to knowing what causes the disease.

Finally, we note that the multiplicative interaction models used for GWAS-based detection of interaction are fundamentally statistical, and thus of necessity assume approximations to the biology. In contrast, our epistatic models directly express what is happening biologically. (E.g., purple flower color in sweet peas, which is DD relative to white; ovoid shape in the seed capsules of sherpherd’s purse, or *Bursa*, which is RR relative to triangular; or feather color in fowl, which is DR relative to white.) This is not a criticism of the statistical models, but it highlights that the statistical models are necessarily approximations to what is happening biologically.

There are many more recently-proposed methods to detect gene-gene interactions from association data [17] such as Bayesian Neural Networks [18], “reduction approaches” [19], and Holzinger et al.’s [16] variable selection method for diseases with very low marginal population risks. These approaches are impressive in their sophistication but still will not overcome the weaknesses discussed above.

### 4.6. Conclusions and future work

Thus, there is ample justification for using the power of linkage analysis to solve genetic problems and determine clearly inherited influences, as opposed to risk factors that may or may not lead to understanding the basic mechanisms of human genetic traits. The current popular genetic research has invested huge resources of time and effort into collecting enormous datasets (10^4^–10^5^ cases and controls), achieving minimal advancement in genetic understanding, while ignoring not only the genetic information (in the form of familial relationships) but also the detailed phenotypic information that has historically been crucial in differentiating different diseases and identifying genetic causes. Such differentiation is crucial to disentangling heterogeneity. In that light, the need to ultimately study families to understand inheritance becomes apparent. Developing and testing robust and powerful methods to detect gene-gene interaction will make the return to family studies more efficacious to genetic investigators.

In this paper we did not set up symmetric hypotheses of “interaction” vs. “heterogeneity.” Rather, we have focused strictly on interaction. The task we set ourselves was to reliably identify interaction when it does exist (EPI and ADD models) and to fail to identify interaction when it does not exist (HET). Our results suggest taking the following approach. First check the *S*_*het*_ > 1 cutoff criterion (Sec. 3.1.) A dataset that does not satisfy that criterion most likely does not result from interaction between two genes. If a dataset meets that criterion, calculate *INT2*. If the statistic is positive, then the dataset most likely does exhibit interaction. If the statistic is negative, that points toward lack of interaction but is not reliable for ADD2 models in which the two genes have different allele frequencies.

As we did in the case of FPAH [3] one could apply the method to the entire genome to search for possible interactions between the known locus and other loci. In future work we will examine theoretical underpinnings that make INT2 such a reliable test statistic for detecting gene-gene interactions, and also study the statistical price to be paid for such whole-genome testing.

We have shown here that our statistic *INT2* can be used to detect evidence of interaction over a broad swath of genetic models with high reliability. These are models in which a disease-related allele at a locus has already been identified or is strongly associated with a marker [4], and where the investigator is trying to determine whether another locus interacts with the first locus. Our method exploits the fact that stratifying family data on the presence of the known disease allele will generally increase the apparent “penetrance” (and hence the LOD scores) if there is interaction, but will not increase the LOD scores otherwise. The *INT2* statistic is a modification of the *INT* statistic studied in our earlier paper [4], and the current work extends that paper.

## Supporting Information

S1 Appendix Formulas for calculating gene frequencies as functions of *r*. **Table A**. Allele frequencies for HET models. **Table B**. Allele frequencies for EPI models. **Table C**. Numerical values for allele frequencies with *r* = 1.
(DOCX)
