# Correction: Using Linkage Analysis to Detect Gene-Gene Interactions. 2. Improved Reliability and Extension to More-Complex Models

**DOI:** 10.1371/journal.pone.0151686

**Published:** 2016-03-11

**Authors:** Susan E. Hodge, Valerie R. Hager, David A. Greenberg

There is an error in the sixth sentence of the first paragraph under the Discussion heading “4.5.2. Issues specific to our study.” The correct sentence is: For example, with our dominant-dominant (DD) model, raising all the not-at-risk genotype penetrances from 0 to 0.01 reduces P to 79%.

There are multiple errors in the two paragraphs below Table 3. The correct paragraphs are:

Define the marginal OR for *A* as $\frac{(p_{1}+p_{2})q_{3}}{(q_{1}+q_{2})p_{3}}$ and the interaction OR as $\frac{p_{1}(q_{2}+q_{3})}{q_{1}(p_{2}+p_{3})}$. Our epistatic models in Fig 2, with all penetrances equaling 1 or 0, lead to ORs of infinity. More interesting is to see what happens when we lower the 1s and raise the 0s. For example, consider a DD model (allele frequencies equal 0.1 for both the *A* and *B* alleles), where we reduce the 1s in Fig 2 to 0.9 and increase the 0s to 0.01. This results in a disease population prevalence of ~4%, and of those affected, ~77% have one of the *AXBX* genotypes (i.e., P = 0.7712). This model is easily handled by our linkage-based methods. It would also be readily detectable by comparing ORs, since the marginal ORs for both the *A* and *B* loci would equal 21.6, and the interaction OR would be 891.

In contrast, consider a model where we reduce the 1s in Fig 2 to 0.4 and raise the 0s to 0.1. The ORs still support interaction: marginal OR = 1.7, interaction OR = 6.0. But now not only is the disease more common (population prevalence ~11%), but of those affected, only 13% have one of the *AXBX* genotypes; 14 have *AX* but not *BX*; another 14% have *BX* but not*AX*; and 59% do not carry either disease allele *A* or *B* at all. Thus, this is more of a heterogeneity, or even nongenetic, model than an interaction model: Many more affected individuals carry either *A* or *B* alone than carry both *A* and *B*, and, further, the overwhelming majority of affected individuals do not carry either disease allele at all. This example illustrates what we said above, that association analysis is more sensitive, yet what it detects may not be particularly helpful for understanding disease causation.
