# Supplementary material for: Using Linkage Analysis to Detect Gene-Gene Interactions. 2. Improved Reliability and Extension to More-Complex Models
Source: PLoS One. 2016 Jan 11;11(1):e0146240. doi: 10.1371/journal.pone.0146240 (PMC4709060; doi:10.1371/journal.pone.0146240)
Supplement: S1 Appendix — Table A. Allele frequencies for HET models. Table B. Allele frequencies for EPI models. Table C. Numerical values for allele frequencies with r = 1. (DOCX) [file pone.0146240.s001.docx]

**Appendix. Formulas for calculating gene frequencies as functions of *r***

*p*_1_ = frequency of *A* allele, *p*_2_ = 1 – *p*_1_

*q*_1_ = frequency of *B* allele, *q*_2_ = 1 – *q*_1_

*K* = population prevalence, *f* = penetrance.

**A.1. HET and EPI models**

For HET models, *K* = .05, *f* = 0.5. For EPI models, *K* = .01, *f* = 1.0.

When the A locus is acting dominantly, its “contribution” is 1 – *p*_2_^2^ ; when recessively, it is *p*_1_^2^ .

Similarly for the B locus: 1 – *q*_2_^2^ for dominant, *q*_1_^2^ for recessive.

Let *r* represent the ratio of A’s contribution to B’s contribution:

$r=\frac{A}{B}$. (A1)

That is, when A is dominant, substitute 1 – *p*_2_^2^ for the numerator in (A1), when dominant, substitute *p*_1_^2^ . When B is dominant, substitute 1 – *q*_2_^2^ for the denominator in (A1); when recessive, substitute *q*_1_^2^ .

Then solve for (*p*_1_, *p*_2_) and (*q*_1_, *q*_2_) .

**HET models**

Prevalence is approximately *K* ≈ *f* × (*A* + *B*) = *f* (*r* + 1)*B*. Make the appropriate substitutions and solve for *B*, then for (*q*_1_, *q*_2_). Then *A* = *rB* , and solve for (*p*_1_, *p*_2_) . See **Table A.**

**Table A. Allele frequencies for HET models**

|  | A locus | B locus |
| --- | --- | --- |
| D+D | $p_{2}=\sqrt{1-\frac{rK}{f(r+1)}}$ , $p_{1}=1-p_{2}$ | $q_{2}=\sqrt{1-\frac{K}{f(r+1)}}$, $q_{1}=1-q_{2}$ |
| D+R | $p_{2}=\sqrt{1-\frac{rK}{f(r+1)}}$ , $p_{1}=1-p_{2}$ | $q_{1}=\sqrt{\frac{K}{f(r+1)}}$ , $q_{2}=1-q_{1}$ |
| R+D | $p_{1}=\sqrt{\frac{rK}{f(r+1)}}$, $p_{2}=1-p_{1}$ | $q_{2}=\sqrt{1-\frac{K}{f(r+1)}}$, $q_{1}=1-q_{2}$ |
| R+R | $p_{1}=\sqrt{\frac{rK}{f(r+1)}}$, $p_{2}=1-p_{1}$ | $q_{1}=\sqrt{\frac{K}{f(r+1)}}$ , $q_{2}=1-q_{1}$ |

**EPI models**

Prevalence is *K* = *f* × (*AB*) , where we specify *f* = 1. Make the appropriate substitutions and solve for *B*, then for (*q*_1_, *q*_2_) . Then *A* = *rB* , and solve for (*p*_1_, *p*_2_) . See **Table B.**

**Table B. Allele frequencies for EPI models**

|  | A locus | B locus |
| --- | --- | --- |
| DD | $p_{2}=\sqrt{1-\left(rK\right)^{1/2}}$ , $p_{1}=1-p_{2}$ | $q_{2}=\sqrt{1-\left(\frac{K}{r}\right)^{1/2}}$, $q_{1}=1-q_{2}$ |
| DR | $p_{2}=\sqrt{1-\left(rK\right)^{1/2}}$ , $p_{1}=1-p_{2}$ | $q_{1}=\left(\frac{K}{r}\right)^{1/4}$ , $q_{2}=1-q_{1}$ |
| RD | $p_{1}=\left(rK\right)^{1/4}$, $p_{2}=1-p_{1}$ | $q_{2}=\sqrt{1-\left(\frac{K}{r}\right)^{1/2}}$, $q_{1}=1-q_{2}$ |
| RR | $p_{1}=\left(rK\right)^{1/4}$, $p_{2}=1-p_{1}$ | $q_{1}=\left(\frac{K}{r}\right)^{1/4}$ , $q_{2}=1-q_{1}$ |

**A.2. Additive 2 model**

For ADD2, define *r* as the ratio of the allele frequencies: *r* = *p*_1_/*q*_1_ .

Prevalence is $K=p_{1}^{2}+q_{1}^{2}-p_{1}^{2}q_{1}^{2}+4p_{1}p_{2}q_{1}q_{2}$. (We set *f* = 1) Substitute *rq*_1_ for *p*_1_ and *q* for *q*_1_ , then solve for *q* in $K=(rq)^{2}+q^{2}-(rq)^{2}q^{2}+4(rq)(1-rq)(q)(1-q)$. Solution is found numerically.

**A.3. Numerical values for allele frequencies with *r* = 1.**

**Table C** gives the allele frequencies resulting from the above calculations, for *r* = 1, the value used for most of our analyses (see Sec. 2.2.2 and 3.3). At each locus, the first value is the disease allele frequency.

**Table C. Numerical values for allele frequencies with *r* = 1**

|  | Model | A locus | B locus |
| --- | --- | --- | --- |
| EPI: *K* = .01, *f* = 1 | DD | (.051, .949) | (.051, .949) |
|  | DR | (.051, .949) | (.316, .684) |
|  | RD | (.316, .684) | (.051, .949) |
|  | RR | (.316, .684) | (.316, .684) |
| HET: *K* = .05, *f* = .5 | D+D | (.025, .975) | (.025, .975) |
|  | D+R | (.025, .975) | (.224, .776) |
|  | R+D | (.224, .776) | (.025, .975) |
|  | R+R | (.224, .776) | (.224, .776) |
| ADD: *K* = .03, *f* = 1 | Add2 | (.074, .926) | (.074, .926) |

*K* = population prevalence, *f* = penetrance
